# Autonomic function test during the COVID-19 pandemic: is it safe and sound?

**DOI:** 10.1007/s10286-021-00776-8

**Published:** 2021-02-02

**Authors:** Pietro Guaraldi

**Affiliations:** IRCCS Istituto delle Scienze Neurologiche di Bologna, UOC Clinica Neurologica NeuroMet, Ospedale Bellaria, via Altura 3, 40139 Bologna, Italy

**Keywords:** COVID-19, SARS-CoV-2, Coronavirus, Autonomic nervous system, Autonomic units, Autonomic function test, Safety protocol

The coronavirus disease 2019 (COVID-19) pandemic has dramatically impacted healthcare systems worldwide, which have been compelled to take drastic measures to reorganize their services in a very short period to tackle this unprecedented medical emergency. After a lull in cases during the summer of 2020, positive rates rose dramatically during the last months of the year, and we are currently facing a second wave even worse than the first. Consequently, the COVID-19 pandemic has changed the way most medical procedures are to be performed in the future, even longer than what we expected.

A challenge for healthcare providers in the provision of adequate medical care is guaranteeing the safety of patients and personnel during routine diagnostic procedures, such as autonomic function testing. Autonomic function testing is essential for detection, quantification, and localization of autonomic dysfunction of several medical conditions, and are relevant to diagnosis, prognosis, and clinical management of patients to improve their quality of life [1]. Furthermore, some have hypothesized that the autonomic nervous system is involved in the pathophysiology of COVID-19 [4, 5], and that autonomic dysfunction could be part of the spectrum of long-term sequelae in survivors, known as “long-haul COVID” [8]. If this is true, referral for autonomic testing would be expected to rise soon. In fact, some cases of orthostatic intolerance after COVID-19 have been published [7, 10], although the causal relationship is yet to be defined.

In this context, the American Autonomic Society (AAS) [3] and the autonomic disorders scientific panel of the European Academy of Neurology [2], in the absence of evidence-based randomized trial data addressing these issues, provided safety guidance for autonomic function testing based on the expert opinion of leaders in the field. Our group also provided some practical advice based on current national and international health authorities’ recommendations and our experience about how to perform cardiovascular autonomic function testing during the COVID-19 emergency [6].

In this special issue of *Clinical Autonomic Research* commemorating its 30th anniversary, Sinn and colleagues share their experience in resuming autonomic function testing at Stanford University [9]. Their safety protocol is similar to the one recommended by the AAS statement. It is also the same as our Bologna group [6]. Some differences include (i) the requirement of polymerase chain reaction (PCR) screening no later than 7 days before the autonomic testing; (ii) the use of both a disposable viral filter and a tube during the Valsalva maneuver; and, (iii) the optional use of disposable gowns. No specific parameters define the time and method to circulate and renew the air in the testing rooms, between one patient and the next. Interestingly, between March 16 and August 2, 2020, they assessed a total of 267 patients, none of which developed COVID-19 immediately after the autonomic testing. This is very good news. The study has limitations acknowledged by the authors. For instance, without repeated PCR tests, they could not completely rule out the possibility of asymptomatic patients. In summary, autonomic function testing is safe and can be resumed as long as it is performed following safety protocols.

Screening with PCR and the ongoing vaccination campaign may help us further, but should never drive the healthcare professional to underestimate the potential risk of dealing with asymptomatic carriers. It is imperative to keep calm, comply with the safety protocol, and perform autonomic function testing when required.
